# Development of deep learning-assisted overscan decision algorithm in low-dose chest CT: Application to lung cancer screening in Korean National CT accreditation program

**DOI:** 10.1371/journal.pone.0275531

**Published:** 2022-09-29

**Authors:** Sihwan Kim, Woo Kyoung Jeong, Jin Hwa Choi, Jong Hyo Kim, Minsoo Chun

**Affiliations:** 1 Department of Applied Bioengineering, Graduate School of Convergence Science and Technology, Seoul National University, Seoul, Republic of Korea; 2 ClariPi Research, Seoul, Republic of Korea; 3 Department of Radiology, Samsung Medical Center, Sungkyunkwan University School of Medicine, Seoul, Republic of Korea; 4 Department of Radiation Oncology, Chung-Ang University College of Medicine, Seoul, Republic of Korea; 5 Center for Medical-IT Convergence Technology Research, Advanced Institutes of Convergence Technology, Suwon, Republic of Korea; 6 Department of Radiology, Seoul National University College of Medicine, Seoul, Republic of Korea; 7 Department of Radiology, Seoul National University Hospital, Seoul, Republic of Korea; 8 Institute of Radiation Medicine, Seoul National University Medical Research Center, Seoul, Republic of Korea; 9 Department of Radiation Oncology, Chung-Ang University Gwang Myeong Hospital, Gyeonggi-do, Republic of Korea; University of Texas at Arlington, UNITED STATES

## Abstract

We propose a deep learning-assisted overscan decision algorithm in chest low-dose computed tomography (LDCT) applicable to the lung cancer screening. The algorithm reflects the radiologists’ subjective evaluation criteria according to the Korea institute for accreditation of medical imaging (KIAMI) guidelines, where it judges whether a scan range is beyond landmarks’ criterion. The algorithm consists of three stages: deep learning-based landmark segmentation, rule-based logical operations, and overscan determination. A total of 210 cases from a single institution (internal data) and 50 cases from 47 institutions (external data) were utilized for performance evaluation. Area under the receiver operating characteristic (AUROC), accuracy, sensitivity, specificity, and Cohen’s kappa were used as evaluation metrics. Fisher’s exact test was performed to present statistical significance for the overscan detectability, and univariate logistic regression analyses were performed for validation. Furthermore, an excessive effective dose was estimated by employing the amount of overscan and the absorbed dose to effective dose conversion factor. The algorithm presented AUROC values of 0.976 (95% confidence interval [CI]: 0.925–0.987) and 0.997 (95% CI: 0.800–0.999) for internal and external dataset, respectively. All metrics showed average performance scores greater than 90% in each evaluation dataset. The AI-assisted overscan decision and the radiologist’s manual evaluation showed a statistically significance showing a *p*-value less than 0.001 in Fisher’s exact test. In the logistic regression analysis, demographics (age and sex), data source, CT vendor, and slice thickness showed no statistical significance on the algorithm (each p-value > 0.05). Furthermore, the estimated excessive effective doses were 0.02 ± 0.01 mSv and 0.03 ± 0.05 mSv for each dataset, not a concern within slight deviations from an acceptable scan range. We hope that our proposed overscan decision algorithm enables the retrospective scan range monitoring in LDCT for lung cancer screening program, and follows an as low as reasonably achievable (ALARA) principle.

## Introduction

Although computed tomography (CT) technology has developed and achieved outstanding diagnostic accuracy, there remains concerns regarding radiation-induced cancers driving an as low as reasonably achievable (ALARA) movement [1–3]. The adoption of a low-dose CT (LDCT) protocol and the scan range optimization are exemplary efforts to lower imaging doses [4–10]. Particularly, LDCT has been popularly used in lung cancer screening program enabling to detect cancers in early stage, thereby reducing mortality rates [11, 12]. While the LDCT protocol has been adopted with various aspects, to the best of our knowledge, scan range selections still rely on a manual decision by the radiation technologist exhibiting intra- and inter-institution variations [13–19]. However, as a manual range selection is vulnerable to prevent excessive patients’ doses, efforts should be made to provide optimal scan range and reduce inter-individual variability. Whereas excessive scan may increase unnecessary doses, the worse scenario is to scan with insufficient coverages requiring additional examination [20–22]. Both situations necessitate the scan range monitoring procedure either prospective or retrospective way.

According to the regulations for the operation of special medical equipment in South Korea, CT scanners have to be inspected every three years in terms of image quality and adequacy of the image acquisition method by an official CT certified agency such as the Korean institute for accreditation of medical imaging (KIAMI) [23]. Among various inspection items, an overscan audit is performed by an expert radiologist by visually inspecting scan ranges whether they are excessive or deficient to the criterion landmarks, which is laborious and subjective. Moreover, this manual auditing process could be performed for the only representative single scan, and the current process could not be applied to all patients’ scan. Fully automated decision program might help to reduce subjectivity, be applied to all CT scans, and save time, human and cost resources. To evaluate the appropriateness of scan ranges, the objective criteria to determine overscan and underscan should be reasonably established according to the regions being scanned and clinical needs. In the audit process for lung cancer screening program in South Korea, the vocal cords and the kidney are used as landmarks for the superior and inferior limit, respectively.

Recent advancements in artificial intelligence (AI) technologies enabled to significantly reduce the time and cost resources of radiologists in various radiology applications, such as lesion classification, detection and segmentation [24–32]. Combining AI technology for organ segmentation and experts’ decision rule-based logical operation, we developed automated overscan decision algorithm in lung cancer screening program and demonstrated the performances with internal and external dataset.

## Materials and methods

### Dataset

A total of 340 LDCT scans for lung cancer screening program was used for decision model development and validation, of which 290 scans were from our institution, named as internal data, and 50 scans were collected from the 47 institutions, named as external data. While the internal data were approved by an institutional review board (IRB) at Seoul National University Hospital (IRB No. 2012-187-1186), no IRB approval was obtained for external data. A submission and the approval of the external data were subject to the National CT Accreditation Program conducted by the KIAMI. For both internal and external data, the informed consent was waived because all CT scans were retrospectively obtained, and all personal information tags in DICOM files were anonymized.

Among 290 internal data, 80 scans were used to develop landmark segmentation model as they have ground truth of landmark structures manually delineated by the expert radiologist with 21 years of experience. For details, 50 CT scans (Siemens 20 cases, GE 10 cases, Philips 20 cases) were used for model training and 10 CT scans (Siemens 4 cases, GE 2 cases, Philips 4 cases) were used for model tuning. The other 20 CT scans (Siemens 8 cases, GE 4 cases, Philips 8 cases) were used for its performance test. All internal data was reconstructed with a vendor-specific iterative reconstruction algorithm. Demographics and scan parameters for the internal data are described in Table 1. Note that the newly introduced tin filter technology was utilized in Siemens Force scanner [33–35].

**Table 1:** Details on internal data. Demographics and scan parameters are presented according to the data usage.

Purpose	Vendor	Scanner name	# of scans (M/F)	Age	kVp	mAs	^a^AEC	Slice thickness (mm)	Reconstruction kernel	Note
Segmentation model development	Siemens	Definition Flash	16	69±9	120	30	Off	1	B60f	Segmentation mask
			(16/0)							
		Force	16	70±11	Sn100^b^	150	Off	1	Br59d\3	
			(14/2)							
	GE	Revolution	16	64±11	120	20±4	On	1.25	Standard	
			(14/2)							
	Philips	Ingenuity	16	67±10	120	30	Off	1	YC	
			(14/2)							
		ICT	16	63±13	120	20	Off	1	YC	
			(14/2)							
Decision model validation	Siemens	Definition flash	30	64±12	120	30	Off	1	I70f\3	Overscan tag
			(17/13)							
		Force	30	61±13	Sn100	150	Off	1	Br59d\3	
			(17/13)							
	GE	Revolution	30	62±13	120	23±3	On	1.25	LUNG	
			(21/9)							
		Discovery	30	61±13	120	42	Off	1.25	LUNG	
			(15/15)							
	Philips	Ingenuity	30	64±14	120	30	Off	1	YC	
			(16/14)							
		IQon	30	65±11	120	20	Off	1	YC	
			(18/12)							
	Canon	Aquilion	30	71±19	120	97±26	On: 8	1	FC15-H	
			(15/15)			30	Off: 22			

^a^AEC: Automatic exposure control,

^b^Sn100: 100 kVp with tin filter attached.

The other 210 internal data and 50 external data were used to demonstrate the performance of the overscan decision model. Fifty external data were obtained from 20 university hospitals and 27 private hospitals, and were consisted of 26 different CT scanners with various low-dose CT scan conditions. Due to their huge diversity, detailed information is presented in S1 Table. All 260 data were marked with overscan tags established by the radiologist’s evaluation. Data from internal and external sources with overscan tagging showed rates of 22.4% and 32%, respectively.

### Overscan decision criteria

**Fig 1:**
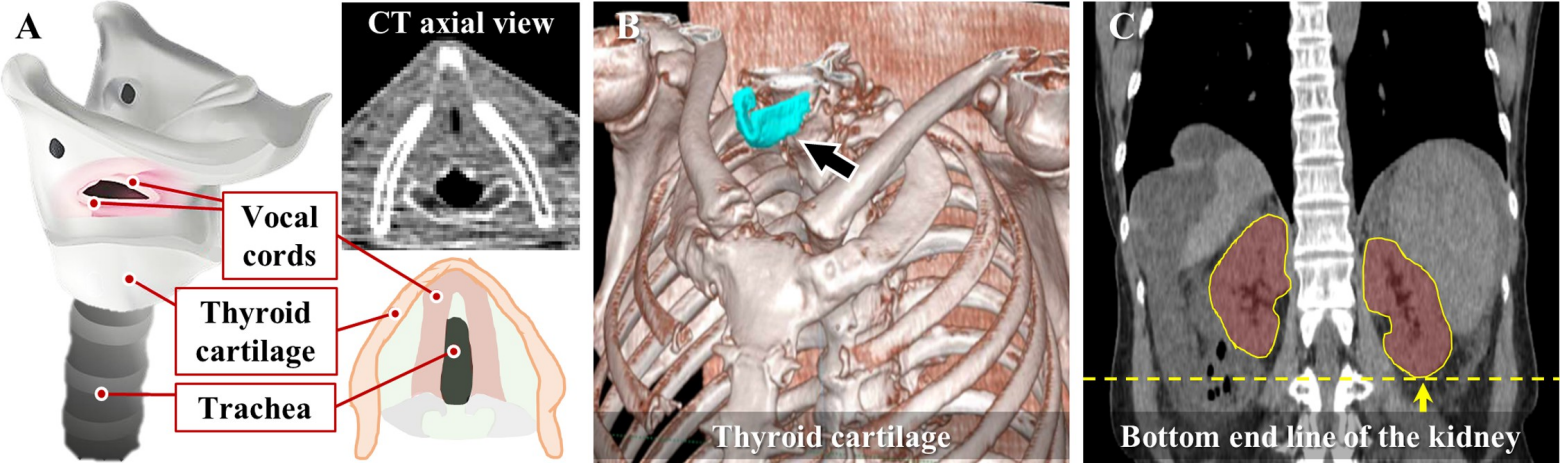
Representative landmarks in overscan decision. (A) Vocal cord as an initial superior-side landmark, (B) Thyroid cartilage as a replaced superior-side landmark, (C) Kidney as an inferior-side overscan landmark.

We applied the KIAMI’s overscan decision criteria in lung cancer screening CT images in the algorithm development [36]. In these criteria, the upper end of the vocal cords and the lower end of the kidney were used as the reference landmarks to represent a superior and inferior scan limit, respectively. However, vocal cords exhibit an irregular anatomical shapes and ambiguity in exact localization even by human observers (Fig 1A). Rather, we determined a thyroid cartilage as a substitute to the vocal cord (Fig 1B) because a thyroid cartilage is easier to be segmented presenting high contrast in CT images and covers the vocal cords in the longitudinal direction [37]. A kidney was used as an inferior-direction landmark (Fig 1C).

### Overscan decision algorithm

**Fig 2:**
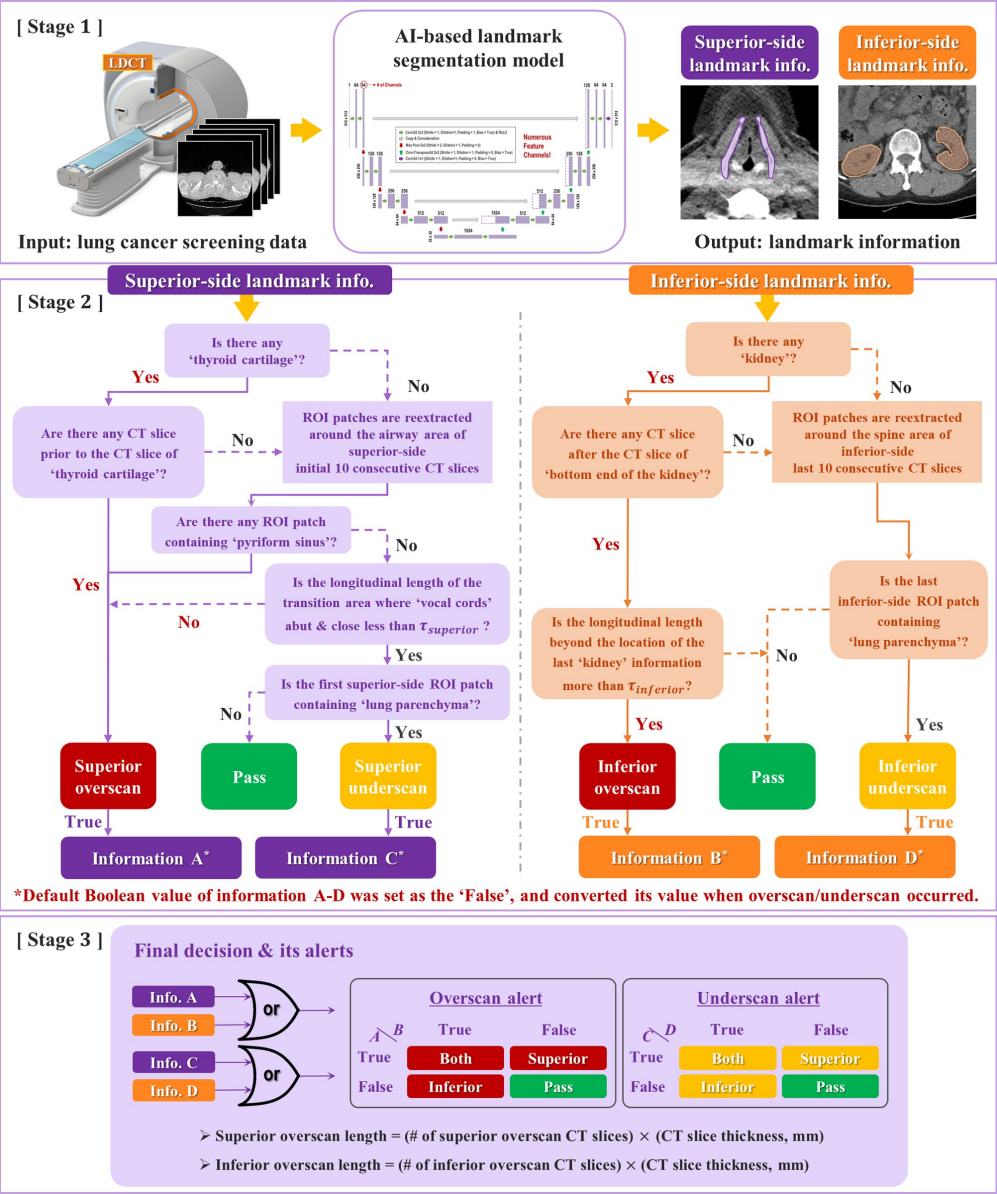
Schematic diagram of overscan determination process. Stage 1: AI-assisted landmark segmentation. Stage 2: Rule-based logical operations. Stage 3: Final overscan decision and its alerts.

The development workflow consisted of three major stages: a deep learning-based fully automated landmark segmentation stages (thyroid cartilage or kidney), the rule-based logical operations for the landmark localization, and the final determination of the overscan direction and its length (Fig 2).

#### AI based landmark segmentation

In the first stage, deep-learning-based fully automated segmentation was implemented using a 2D-image based U-Net model [38]. Key imaging features of the target object were automatically extracted by using concatenated encoding-decoding architecture. The model was trained by using a supervised learning with manually labelled landmarks. For efficient parameter learning with relatively small data, they were randomly augmented by rotation (within 5 degrees) and translation (within 5 pixels) in each training iteration. In addition, the binary cross-entropy loss for two classes (one for the background and the other for the target object) was optimized with an iterative learning process, and an optimal training iteration was determined by an early stopping method using the AI-model tuning dataset [39]. To maximize the performance, two separate U-Net based segmentation models were trained for each landmark. When the lung cancer screening data were imported to the model, landmark’s binary masks were generated and passed to the rule-based logical operation stage.

#### Sub-algorithm for rule-based logical operation

In the second stage, there are two determination branches according to the landmark types. Each branch comprised of rule-based logical operations, reflecting the human decision-making process. The identification information (information A-D in Fig 2) of overscan/underscan was logically acquired for each determination branch, based on the information of predefined landmark presence and its relative position to initial/last CT slice.

When the thyroid cartilage was localized, the existence of images prior to the thyroid cartilage was examined to decide the superior-side overscan. If the thyroid cartilage was not detected, ROI patches were scanned for the ten-consecutive superior slices around the airway region to cope with false-negative cases of the landmark. The ROI patches had 64 × 64 pixels and the airway region was localized by thresholding CT numbers from -1000 HU to -900 HU. The algorithm decided the overscan when the ROI patches detected the pyriform sinus. In case of no pyriform sinus in the patch, the length of vocal cords being touched each other or closed are examined. While the overscan decision was made when the length exceeded the threshold (τ_*superior*_), the algorithm further examined whether the patches contained the lung parenchymal tissue if the length was less than the threshold. The decision was made as appropriate scan range or underscan according to the existence of the lung parenchymal tissue within the patches. The optimal τ_*superior*_ was empirically obtained by using the internal data.

Similarly, in inferior-side determination, the inferior-side overscan was decided with relative location of the landmark organ, kidney. To cope with a real absence of the kidney or prevent false-negative cases, ROI patches with a 64 × 64-pixel area were extracted around the spine region within the ten-consecutive inferior-side CT slices. The spine region was detected by thresholding CT numbers of 150 HU. If the range of CT scanning exceeded the location of the last kidney, an inferior overscan was determined accordingly. An optimal inferior-side threshold value (τ_*inferior*_) for the exceeding length of the inferior-side scan was determined by referencing a possible movement range of the kidney by intrinsic human motion (e.g., breathing or peristalsis of digestive organs). As in the case of the superior underscan, the information on lung parenchyma presence was used to filter out the inferior underscan. If the last inferior-side ROI patch included the region of the lung parenchyma, it was concluded as the inferior underscan. Scan range identification information (Fig 2, information A-D), as the results for logical operation stage, were set as ‘False’ at default. When the overscan or underscan occurred, the Boolean value was converted from ‘False’ to ‘True’. The final Boolean values in information A-D were transmitted to the last decision stage.

#### Final decision and its alerts

In the last stage, the final determination of the overscan direction and its length was made through the identification information resulted from rule-based logical operations for both superior-side and inferior-side. When the identification information on the overscan (Fig 2, information A or information B) and the underscan (Fig 2, information C or information D) were given as the Boolean-type data (e.g. ‘True’ or ‘False’), the overscan/underscan direction was determined by the logical OR gate. After finishing each detection process, the algorithm alerted scan range status. While the underscan detection is limited to alerting the scan status, overscan detection further includes the overscan length calculation. The overscan length was calculated by multiplying the slice thickness of the reconstructed CT image and the number of slices determined as the overscan.

### Evaluation design

#### Segmentation model evaluation

The segmentation performance was evaluated with dice similarity coefficient (DSC) for each patient [40]. The DSC ranged from 0 to 1 corresponding to no-overlap and complete overlap, respectively. The evaluation was with AI-model tuning and test data of the development dataset for thyroid cartilage and kidney, respectively. Mean and standard deviation of DSC for those data were calculated.

#### Optimal threshold cut-off

The optimal τ_*superior*_ was heuristically determined as 9 mm according to the receiver operating characteristic (ROC) analysis with internal data. In contrast, the τ_*inferior*_ was determined as 3 mm by considering the intrinsic movement range of the kidney [41].

#### Metrics for decision model validation

The area under the receiver operating characteristic (AUROC) metric was used to assess the overall algorithm performance with the linear approximation of ROC curves. At the given optimal τ_*superior*_ and τ_*inferior*_, the accuracy, sensitivity, and specificity were calculated by using the standard logit method, and their corresponding 95% confidence intervals (CI) were calculated with the Clopper-Pearson’s method [42–45]. The Cohen’s kappa was calculated by using McHugh’s formula [46]. All metrics ranged from 0 to 1 and are expressed as percentages except for kappa and AUROC. Fisher’s exact test was used to test whether the developed algorithm could distinguish overscan cases [47]. A significance level was set at 0.05. All performance metrics were calculated by using MedCalc software (Version 20.023, MedCalc Software Ltd., Mariakerke, Belgium).

#### Evaluation procedure

First, the AUROC was calculated using each decision model validation data, and the optimal threshold was selected as a cut-off value under the best algorithm performance while maintaining conservative determination criteria (e.g., the shorter the CT scan range became, the lower the radiation exposure that could be achieved.) through ROC analysis using the internal data. Second, based on the selected cut-off value, as evaluation metrics, accuracy, sensitivity, and specificity were calculated on the validation data. Finally, a univariate logistic regression analysis was used to assess the generalizability of the developed algorithm, as well as its accuracy on each independent variable [48].

#### Univariate logistic regression analysis

The univariate logistic regression was performed to demonstrate statistical significances of the algorithm according to the patient’s characteristics (e.g. age and sex), data source, CT vendor, and slice thickness. The analysis was conducted based on the confusion matrix of rearranged data with respect to the five independent variables using both internal and external data (S2 Table). The dependent variable was the correctness of the overscan decision. It evaluated the accuracy with respect to the components of each variable and calculated their odds ratios (ORs) with 95% CIs. The IBM SPSS Statistics software (version 25.0; IBM Corp., Armonk, NY, USA) was used, and the statistical significance was set as *p*-value < 0.05.

#### Excessive effective dose estimation for overscan

The excessive effective dose was estimated by multiplying volume CT dose index (CTDI_vol_), overscan length and the tissue conversion factor (*k*). The CTDI_vol_ was obtained from either CT DICOM header or a structured dose report. The *k* value was referenced from the report of American Association of Physicists in Medicine (AAPM) and was 0.0059 and 0.015 for the superior and inferior-side overscan, respectively, which corresponds to neck and abdomen region [49]. The detailed scan parameters and the excessive effective dose was provided in S3 Table.

## Results

### Segmentation model evaluation

**Fig 3:**
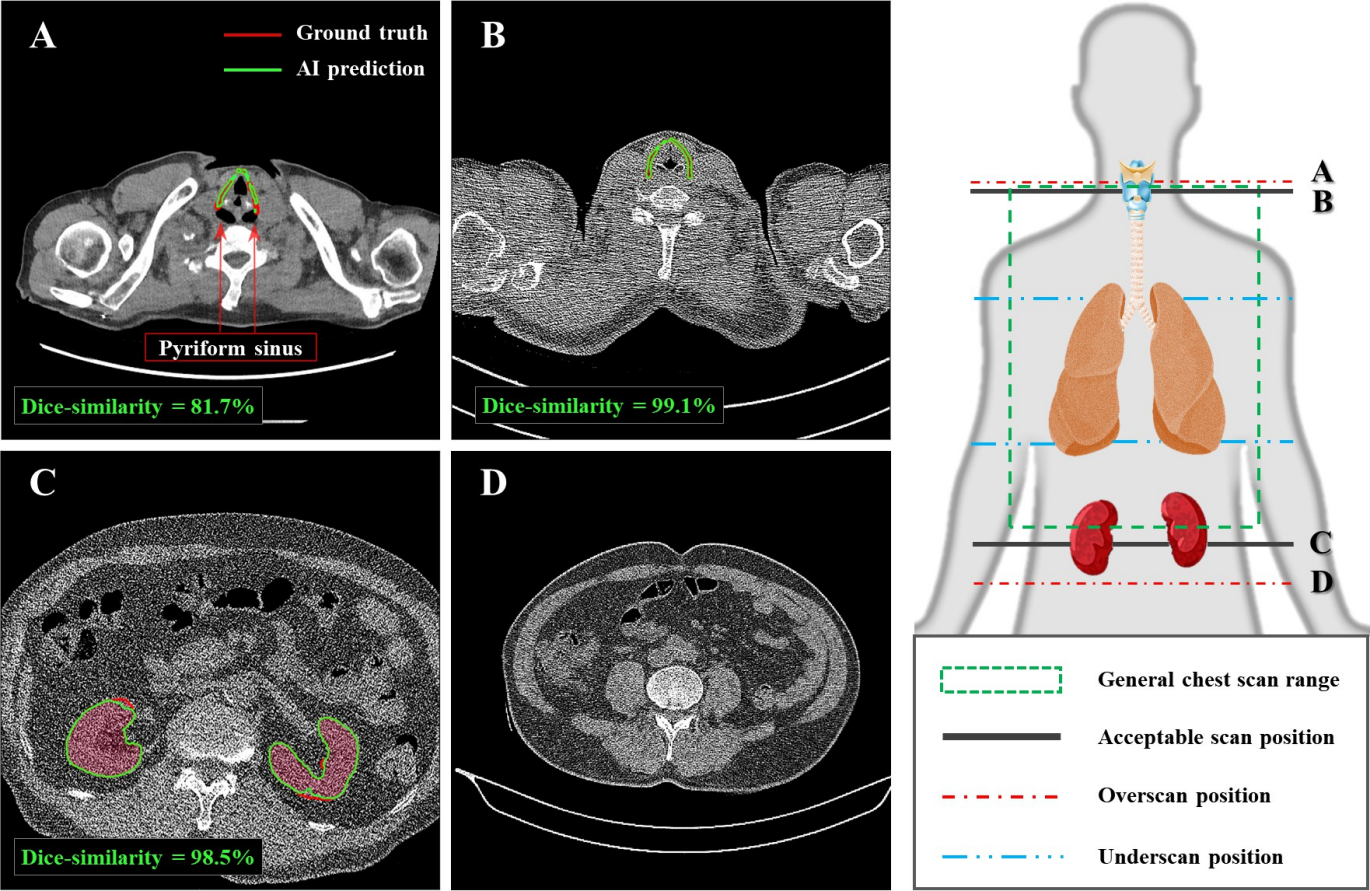
Sample landmark segmentation comparison between AI-assisted model and the experienced radiologist. The results were visualized at different scanning position; (A) segmentation result of superior-side scan at overscan position, (B) that of superior-side scan at acceptable scan position, (C) that of inferior-side scan at acceptable scan position, and (D) inferior-side scanning example at overscan position. Ground truth label and AI-model’s prediction result were displayed with red and green lines, respectively. (W/L: 400/40).

The DSCs of landmark segmentations on LDCT AI-model tuning data were 0.76 ± 0.09 and 0.88 ± 0.14 for thyroid cartilage and kidney, respectively. On LDCT testing data, the DSCs for thyroid cartilage and kidney were 0.79 ± 0.25 and 0.93 ± 0.09, respectively. Except for slices of the superior-side overscan (Fig 3A), the AI-assisted segmentation showed DSCs over 90% with manual delineation within a general scan range (Fig 3B and 3C). In the superior-side scanning, 90% of the overscan data were scanned up to position of the pyriform sinus level.

### Decision model validation

**Fig 4:**
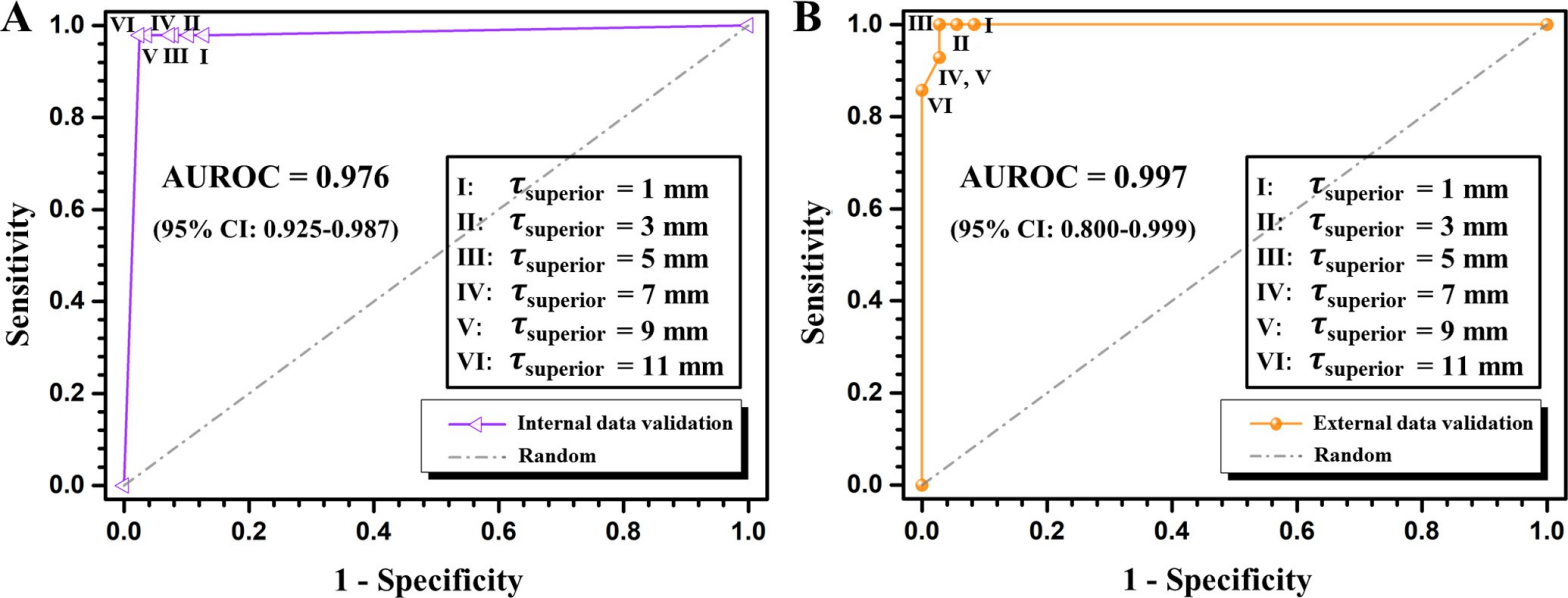
ROC curve and AUROC values for internal (A) and external data (B), respectively.

Fig 4 shows the ROC curves of the overscan decision algorithm. The AUROC values were 0.976 (95% CI: 0.925–0.987) and 0.997 (95% CI: 0.800–0.999) for the internal and external data, respectively. For the optimal *τ*_*superior*_ and *τ*_*inferior*_, calculating from 2x2 contingency table (Table 2), all evaluation metrics showed greater than 90%. In decision model validation, the accuracy of the algorithm was 96.67 (95% CI: 93.25%-98.65%) and 96.00 (95% CI: 86.29%-99.51%) for internal and external data, respectively. While the sensitivity was shown as 97.87% (95% CI: 88.71%-99.95%) and 93.75% (95% CI: 69.77%-99.84%), the specificities were 96.32% (95% CI: 92.16%-98.64%) and 97.06% (95% CI: 84.67%-99.93%) for internal and external data, respectively. The Fisher’s exact test between our decision algorithm and the experienced radiologist showed statistical significances (*p* < 0.001).

**Table 2:** Contingency table of decision model validation with internal and external data.

Contingency table of internal data		Gold standard by an audit-experienced radiologist	
		Overscan	None
Algorithm prediction	Overscan	46	6
	None	1	157
Contingency table of external data		Gold standard by an audit-experienced radiologist	
		Overscan	None
Algorithm prediction	Overscan	15	1
	None	1	33

### Univariate logistic regression analysis

Table 3 shows the results of univariate logistic regression analysis. The univariate analysis revealed that age, sex, data source, CT vendor, and slice thickness had no statistically significant influence on the algorithm’s decision. The individual accuracy of the algorithm for the rearranged data according to the five independent variables was above 95%.

**Table 3:** Univariate logistic regression analysis. The internal and external data were rearranged to satisfy pre-defined univariate condition for each variable.

Independent variable		Univariate analysis			Performance	
		OR^a^	95% CI^b^	p-value	Accuracy (%)	95% CI
**Age (n = 210)**	**age ≤ 64 yr (n = 106)**	1	-	-	98.1	[93.4, 99.8]
	**age > 64 yr (n = 104)**	0.381	[0.07, 2.01]	0.255	95.2	[89.1, 98.4]
**Sex (n = 210)**	**Male (n = 119)**	1	-	-	95.8	[90.5, 98.6]
	**Female (n = 91)**	1.952	[0.37, 10.30]	0.431	97.8	[92.3, 99.7]
**Data source (n = 260)**	**Internal (n = 210)**	1	-	-	96.7	[93.3, 98.7]
	**External (n = 50)**	0.828	[0.17, 4.11]	0.817	96.0	[86.3, 99.5]
**CT vendor (n = 260)**	**Siemens (n = 83)**	1	-	-	96.4	[89.8, 99.3]
	**GE (n = 71)**	0.85	[0.17, 4.35]	0.845	95.8	[88.1, 99.1]
	**Philips (n = 65)**	1.181	[0.19, 7.29]	0.858	96.9	[89.3, 99.6]
	**Canon (n = 41)**	1.5	[0.15, 14.88]	0.729	97.6	[87.1, 99.9]
**Slice thickness (mm, n = 260)**	**1 ≤ t < 2 (n = 238)**	1	-	-	96.6	[93.5, 98.5]
	**2 ≤ t ≤ 5 (n = 22)**	0.73	[0.09, 6.12]	0.772	95.5	[77.2, 99.9]

^a^OR: Odds ratio,

^b^CI: Confidence interval

### Excessive effective dose estimation for overscan

Detailed scan parameters and estimated excessive effective doses for overscan data were provided in S3 Table. The mean CTDI_vol_ of overscan cases was 2.42 ± 1.69 mGy and 2.54 ± 0.65 mGy, and the mean overscan length was 1.59 ± 0.7 cm and was 1.66 ± 1.41 cm for internal and external data, respectively. The estimated effective doses caused by the overscan increased about 0.02 ± 0.01 mSv and 0.03 ± 0.05 mSv for internal and external data, respectively.

## Discussion

The purpose of our study was to develop a deep learning-assisted algorithm to discriminate the overscan cases of lung cancer screening program in LDCT scan. Most of the previous studies on overscan determination [10, 18] in chest CT scans mainly focused on scan range delimitation in topograms, but not in axial CT slices. Automatic scan range delimitation in the topogram domain would be a meaningful approach because the topogram defined the regions being scanned prior to actual scans. In these regards, scan range recommendations in topogram stage enable to reduce the workload and inter-operator variability, and there have been various related researches. However, it is challenging and premature because there are opaque shadings and intensity variation across CT vendors in topograms, and even AI-assisted approaches are still hard to localize key landmarks [22]. Furthermore, localization comparison between scout and axial images should be preceded for their clinical routine [50]. Although optimal scan range recommendations prior to CT scans is meaningful, we mainly focused to establish more objective system to retrospectively assess the overscan length and its corresponding excessive doses, and there are no previous related studies.

Our proposed algorithm presented remarkable performances showing values greater than 95% and 97% of accuracy and AUROC, respectively. The developed algorithm showed almost perfect agreement with experienced radiologists, with kappa values of 0.907 and 0.908 for internal and external dataset, respectively. Furthermore, the Fisher’s exact test demonstrated that the proposed algorithm had an ability to detect the overscan similar to the audit-experienced experts. The logistic regression analysis showed no statistical significances with various potential variables in LDCT, such as age, sex, data source, CT vendor, and slice thickness. These results suggest that the algorithm has a decision-making ability similar to that of a radiologist, and it has a high potential for generalizability in LDCT. In other words, despite differences in LDCT protocols between hospitals, the proposed algorithm could be generalized for the majority of patients taking lung cancer screening in LDCT, and this can be applicable to hospitals’ independent quality control practice.

Considering the mean effective dose being about 0.02 mSv in case of plain PA chest radiograph, the estimated excessive effective dose in our study are equivalent or 1.5 times greater than those in chest PA on average [51]. In the worst overscan case of the study, the excessive effective dose caused by overscan could reach up to 11 times of those by plain PA chest radiograph. However, all those effective dose levels were much below the possible chromosomal damage level (5 mSv) by the X-ray radiation [52]. In the case of slight deviations from the acceptable scan range, especially for LDCT scan, it is thought that excessive effective dose is not a concern.

This study had a few limitations. First, a subjective bias could affect the gold standard, even though it was created by an experienced radiologist. In the future study, it might better to secure an objectivity by utilizing records of more than two experts. Second, in the AI model segmentation stage, there might exist segmentation errors for both superior and inferior direction (S1 Fig). The main reasons were extreme quantum-noise of LDCT and lack of a diversity of trained data distribution. However, despite the segmentation errors, the developed algorithm minimized its vulnerability to error cases by aggregating the 2D segmented landmark information into 3D volumetric information. Furthermore, several segmentation errors could be filtered out and improved by the logical operation of the second-stage. Third, analyzing the contingency table in both internal and external data, the algorithm still had a few false-negatives (algorithm = none, radiologist = overscan) and false-positives (algorithm = overscan, radiologist = none). The reason for false-negative occurrences in both data was that the length of the transition area in superior-side scan was not over the pre-defined overscan cut-off threshold. In most of the false-positive cases, the partial segmentation failure of the landmark at the initial or last slice position was considered as a main reason in our algorithm. Although our algorithm could coordinate false-positives and false-negatives by adjusting the cut-off threshold (τ_*superior*_ or τ_*inferior*_), the optimal threshold might require a large scale and multi-centre investigation. Fourth, we could not prospectively suggest the optimal scan range prior to CT scan while other topogram-based approaches did. As stated above, the further thorough demonstration of its clinical appropriateness between topogram and axial slices retrospectively with large amount of dataset enable to reliably suggest the optimal scan range, thereby achieve fully automated scan range suggestion practice in lung cancer screening. Lastly, there exists only few underscan cases (0.4%), and the separate study to evaluate the underscan is necessary as a future research topic. Also, the demonstrations were limited to LDCT, it could be applied to standard-dose CT as applications work better in standard dose CT than LDCT.

Beyond limitations, by using the developed algorithm, the radiologists only need to pay attention to the alerting cases by the overscan/underscan. The less overscan rate, the less radiologist’s workload could be accomplished. In comparison with every single patient audit procedure, as the overscan occurred in internal and external datasets at a frequency of 22.4% and 32%, it was estimated that the developed algorithm could reduce the workload of overscan range check by 68% and 77.6% for each dataset. We also expect that combinations of our algorithm with radiation dose and image quality monitoring could establish the fully automated and integrated CT quality system for every patient [53–57]. By reducing human and time resources with full automation, it is available to equip high throughput and objective quality monitoring platform for the entire CT scans.

## Conclusions

We developed the deep learning-assisted overscan decision algorithm including three stages of AI-based landmark segmentation model, rule-based logical operation, and final determination. In the demonstration with 210 and 50 lung cancer screening cases for internal and external dataset, the algorithm showed values of greater than 96.0% and 97.6% of accuracy and AUROC, respectively. For LDCT chest screening, the excessive effective dose is not a concern within the slight deviations from the acceptable scan range. We hope that the combination of the proposed algorithm along with multi-parametric image quality assessment, and radiation dose monitoring program will lead the scan range and protocol optimization, and contribute patients’ radiation safety by following ALARA principle. Furthermore, hospitals enable to establish an independent quality monitoring platform and this automated system will allow high throughput and objective quality control to improve the entire CT practice.

## Supporting information

S1 Table Detailed scan parameters for external data. (DOCX)

S2 Table Confusion matrix of each variable in univariate logistic analysis. The variable consists of age, sex, data source, slices thickness and CT vendors. “Correct” and “Incorrect” indicate the success and fail of overscan detection, respectively.
(DOCX)

S3 Table Excessive effective doses caused by overscans for internal and external dataset. (DOCX)

S1 Fig Error examples for AI-assisted segmentation model. Segmentation results for superior-side (A-C) and inferior-side (D-F) were visualized in 2D axial view and 3D rendering view. The use of only two-dimensional information on landmarks’ existence may exhibit an entire (B) or partial (E) segmentation failure, while the use of volumetric information stacked from 2D information has been improved in landmark detections (C, F). Ground truth label and AI predictions were marked as red and green lines, respectively. (W/L = 400/40 HU).
(TIF)

## References

[1] Sodickson A, Baeyens PF, Andriole KP, Prevedello LM, Nawfel RD, Hanson R, et al. Recurrent CT, cumulative radiation exposure, and associated radiation-induced cancer risks from CT of adults. Radiology. 2009; 251(1): 175–84. doi: 10.1148/radiol.2511081296 . 19332852

[2] Shah D, Sachs R, Wilson D. Radiation-induced cancer: a modern view. Br J Radiol. 2012; 85(1020): e1166–e73. doi: 10.1259/bjr/25026140 23175483 PMC3611719

[3] Miglioretti DL, Johnson E, Williams A, Greenlee RT, Weinmann S, Solberg LI, et al. The use of computed tomography in pediatrics and the associated radiation exposure and estimated cancer risk. JAMA Pediatr. 2013; 167(8): 700–7. doi: 10.1001/jamapediatrics.2013.311 . 23754213 PMC3936795

[4] Lee W-J, Ahn B-S, Park Y-S. Radiation dose and image quality of low-dose protocol in chest CT: Comparison of standard-dose protocol. J Radiat Prot Res. 2012; 37(2): 84–9. 10.14407/jrp.2012.37.2.084.

[5] Sagara Y, Hara AK, Pavlicek W, Silva AC, Paden RG, Wu Q. Abdominal CT: comparison of low-dose CT with adaptive statistical iterative reconstruction and routine-dose CT with filtered back projection in 53 patients. AJR Am J Roentgenol. 2010; 195(3): 713–9. doi: 10.2214/AJR.09.2989 . 20729451

[6] Kubo T, Ohno Y, Nishino M, Lin P-J, Gautam S, Kauczor H-U, et al. Low dose chest CT protocol (50 mAs) as a routine protocol for comprehensive assessment of intrathoracic abnormality. Eur J Radiol Open. 2016; 3: 86–94. doi: 10.1016/j.ejro.2016.04.001 . 27957519 PMC5144113

[7] Poletti P-A, Platon A, Rutschmann OT, Schmidlin FR, Iselin CE, Becker CD. Low-dose versus standard-dose CT protocol in patients with clinically suspected renal colic. AJR Am J Roentgenol. 2007; 188(4): 927–33. doi: 10.2214/AJR.06.0793 . 17377025

[8] Niemann T, Kollmann T, Bongartz G. Diagnostic performance of low-dose CT for the detection of urolithiasis: a meta-analysis. AJR Am J Roentgenol. 2008; 191(2): 396–401. doi: 10.2214/AJR.07.3414 . 18647908

[9] Zinsser D, Maurer M, Do P-L, Weiß J, Notohamiprodjo M, Bamberg F, et al. Reduced scan range abdominopelvic CT in patients with suspected acute appendicitis-impact on diagnostic accuracy and effective radiation dose. BMC Med Imaging. 2019; 19(1): 1–7. doi: 10.1186/s12880-019-0304-x . 30635023 PMC6329115

[10] Demircioğlu A, Kim M-S, Stein MC, Guberina N, Umutlu L, Nassenstein K. Automatic Scan Range Delimitation in Chest CT Using Deep Learning. Radiol Artif Intell. 2021; 3(3): e200211. doi: 10.1148/ryai.2021200211 . 34136818 PMC8204132

[11] Kim HY. National lung cancer screening in Korea: introduction and imaging quality control. J Korean Soc Radiol. 2019; 80(5): 826–36. 10.3348/jksr.2019.80.5.826.

[12] Oudkerk M, Liu S, Heuvelmans MA, Walter JE, Field JK. Lung cancer LDCT screening and mortality reduction—evidence, pitfalls and future perspectives. Nat Rev Clin Oncol. 2021; 18(3): 135–51. doi: 10.1038/s41571-020-00432-6 . 33046839

[13] Trattner S, Pearson GD, Chin C, Cody DD, Gupta R, Hess CP, et al. Standardization and optimization of CT protocols to achieve low dose. J Am Coll Radiol. 2014; 11(3): 271–8. doi: 10.1016/j.jacr.2013.10.016 . 24589403 PMC3969855

[14] Yu L, Liu X, Leng S, Kofler JM, Ramirez-Giraldo JC, Qu M, et al. Radiation dose reduction in computed tomography: techniques and future perspective. Imaging Med. 2009; 1(1): 65. doi: 10.2217/iim.09.5 . 22308169 PMC3271708

[15] Ohno Y, Takenaka D, Kanda T, Yoshikawa T, Matsumoto S, Sugihara N, et al. Adaptive iterative dose reduction using 3D processing for reduced-and low-dose pulmonary CT: comparison with standard-dose CT for image noise reduction and radiological findings. AJR Am J Roentgenol. 2012; 199(4): W477–W85. doi: 10.2214/AJR.11.8275 . 22997397

[16] Kim HJ, Park SY, Park YH, Chang AR. Dosimetric Effects of Low Dose 4D CT Using a Commercial Iterative Reconstruction on Dose Calculation in Radiation Treatment Planning: A Phantom Study. Prog Med Phys. 2017; 28(1): 27–32. 10.14316/pmp.2017.28.1.27.

[17] Colevray M, Tatard-Leitman V, Gouttard S, Douek P, Boussel L. Convolutional neural network evaluation of over-scanning in lung computed tomography. Diagn interv imaging. 2019; 100(3): 177–83. doi: 10.1016/j.diii.2018.11.001 . 30497958

[18] Cohen SL, Ward TJ, Makhnevich A, Richardson S, Cham MD. Retrospective analysis of 1118 outpatient chest CT scans to determine factors associated with excess scan length. Clin imaging. 2020; 62: 76–80. doi: 10.1016/j.clinimag.2019.11.020 . 32200203 PMC7598945

[19] Schwartz F, Stieltjes B, Szucs-Farkas Z, Euler A. Over-scanning in chest CT: comparison of practice among six hospitals and its impact on radiation dose. Eur J Radiol. 2018; 102: 49–54. doi: 10.1016/j.ejrad.2018.03.005 . 29685544

[20] Johnson JN, Hornik CP, Li JS, Benjamin DK Jr, Yoshizumi TT, Reiman RE, et al. Cumulative radiation exposure and cancer risk estimation in children with heart disease. Circulation. 2014; 130(2): 161–7. doi: 10.1161/CIRCULATIONAHA.113.005425 . 24914037 PMC4103421

[21] Fabritius G, Brix G, Nekolla E, Klein S, Popp HD, Meyer M, et al. Cumulative radiation exposure from imaging procedures and associated lifetime cancer risk for patients with lymphoma. Sci Rep. 2016; 6(1): 1–9. doi: 10.1038/srep35181 . 27748377 PMC5066222

[22] Kuo P-L, Wu Y-J, Wu F-Z. Pros and Cons of Applying Deep Learning Automatic Scan-Range Adjustment to Low-Dose Chest CT in Lung Cancer Screening Programs. Acad Radiol. 2022. doi: 10.1016/j.acra.2022.02.017 . 35410801

[23] Ministry of Health and Welfare, Republic of Korea. Rules for the installation and operation of special medical equipment, Ordinance No. 817, Published July 7, 2021 [cited 2021 Dec 30]. Available from: https://glaw.scourt.go.kr/wsjo/lawod/sjo190.do?contId=3261196#1663034913233.

[24] Esteva A, Chou K, Yeung S, Naik N, Madani A, Mottaghi A, et al. Deep learning-enabled medical computer vision. NPJ Digit Med. 2021; 4(1): 1–9. doi: 10.1038/s41746-020-00376-2 . 33420381 PMC7794558

[25] Aggarwal R, Sounderajah V, Martin G, Ting DS, Karthikesalingam A, King D, et al. Diagnostic accuracy of deep learning in medical imaging: a systematic review and meta-analysis. NPJ Digit Med. 2021; 4(1): 1–23. doi: 10.1038/s41746-021-00438-z . 33828217 PMC8027892

[26] Cullell-Dalmau M, Noé S, Otero-Viñas M, Meić I, Manzo C. Convolutional Neural Network for Skin Lesion Classification: Understanding the Fundamentals Through Hands-On Learning. Front Med. 2021; 8: 213. doi: 10.3389/fmed.2021.644327 . 33748163 PMC7969634

[27] Wu P, Sun X, Zhao Z, Wang H, Pan S, Schuller B. Classification of lung nodules based on deep residual networks and migration learning. Comput Intell Neurosci. 2020; 2020. doi: 10.1155/2020/8975078 . 32318102 PMC7149413

[28] Yang H, Yu H, Wang G. Deep learning for the classification of lung nodules. arXiv preprint arXiv:161106651. 2016.

[29] Zhang S, Xu S, Tan L, Wang H, Meng J. Stroke Lesion Detection and Analysis in MRI Images Based on Deep Learning. J Healthc Eng. 2021; 2021. 10.1155/2021/5524769.

[30] Kijowski R, Liu F, Caliva F, Pedoia V. Deep learning for lesion detection, progression, and prediction of musculoskeletal disease. J Magn Reson Imaging. 2020; 52(6): 1607–19. doi: 10.1002/jmri.27001 . 31763739 PMC7251925

[31] Isensee F, Jaeger PF, Kohl SA, Petersen J, Maier-Hein KH. nnU-Net: a self-configuring method for deep learning-based biomedical image segmentation. Nat Methods. 2021; 18(2): 203–11. doi: 10.1038/s41592-020-01008-z . 33288961

[32] Liu C-F, Hsu J, Xu X, Ramachandran S, Wang V, Miller MI, et al. Deep learning-based detection and segmentation of diffusion abnormalities in acute ischemic stroke. Commun Med. 2021; 1(1): 1–18. doi: 10.1038/s43856-021-00062-8 . 35602200 PMC9053217

[33] Haubenreisser H, Meyer M, Sudarski S, Allmendinger T, Schoenberg SO, Henzler T. Unenhanced third-generation dual-source chest CT using a tin filter for spectral shaping at 100 kVp. Eur J Radiol. 2015; 84(8): 1608–13. doi: 10.1016/j.ejrad.2015.04.018 . 26001437

[34] Suntharalingam S, Allmendinger T, Blex S, Al-Bayati M, Nassenstein K, Schweiger B, et al. Spectral beam shaping in unenhanced chest CT examinations: a phantom study on dose reduction and image quality. Acad Radiol. 2018; 25(2): 153–8. doi: 10.1016/j.acra.2017.08.011 . 29055683

[35] Zhang W-G, Liu J-P, Jia X-Q, Zhang J-Y, Li X-N, Yang Q. Effects of the Sn100 kVp Tube Voltage Mode on the Radiation Dose and Image Quality of Dual-Source Computed Tomography Pulmonary Angiography. Int J Gen Med. 2021; 14: 1033. doi: 10.2147/IJGM.S293173 . 33790632 PMC8006964

[36] Korea Institute of Associated Medical Imaging (KIAMI) [Internet]. Standard guideline for low dose chest CT scan. [cited 2021 Dec 30] Available from: http://kiami.or.kr/Kiami/Default.aspx?lm=3&rp=38.

[37] Cinar U, Yigit O, Vural C, Alkan S, Kayaoglu S, Dadas B. Level of vocal folds as projected on the exterior thyroid cartilage. Laryngoscope. 2003; 113(10): 1813–6. doi: 10.1097/00005537-200310000-00028 . 14520111

[38] Ronneberger O, Fischer P, Brox T, editors. U-net: Convolutional networks for biomedical image segmentation. Med Image Comput Comput Assist Interv; 2015.

[39] Morgan N, Bourlard H, editors. Generalization and parameter estimation in feedforward nets: Some experiments. Adv Neural Inf Process Syst; 1989.

[40] Dice LR. Measures of the amount of ecologic association between species. Ecology. 1945; 26(3): 297–302. 10.2307/1932409.

[41] Siva S, Pham D, Gill S, Bressel M, Dang K, Devereux T, et al. An analysis of respiratory induced kidney motion on four-dimensional computed tomography and its implications for stereotactic kidney radiotherapy. Radiat Oncol. 2013; 8(1): 1–8. doi: 10.1186/1748-717X-8-248 . 24160868 PMC3829388

[42] Clopper CJ, Pearson ES. The use of confidence or fiducial limits illustrated in the case of the binomial. Biometrika. 1934: 404–13. https://doi.org/2331986.

[43] Trevethan R. Sensitivity, specificity, and predictive values: foundations, pliabilities, and pitfalls in research and practice. Front Public Health. 2017; 5: 307. doi: 10.3389/fpubh.2017.00307 . 29209603 PMC5701930

[44] Altman D, Machin D, Bryant T, Gardner M. Statistics with confidence: confidence intervals and statistical guidelines: John Wiley & Sons; 2013.

[45] International Organization for Standardization (ISO) [internet]. Accuracy (trueness and precision) of measurement methods and results—Part 1: General principles and definitions, ISO 5725–1:1994(en) [cited 2021 Dec 31]. Available from: https://www.iso.org/obp/ui/#iso:std:iso:5725:-1:ed-1:v1:en.

[46] McHugh ML. Interrater reliability: the kappa statistic. Biochem Med. 2012; 22(3): 276–82. . 23092060 PMC3900052

[47] Fisher RA. On the interpretation of χ2 from contingency tables, and the calculation of P. J R Stat Soc. 1922; 85(1): 87–94. 10.2307/2340521.

[48] Tolles J, Meurer WJ. Logistic regression: relating patient characteristics to outcomes. JAMA. 2016; 316(5): 533–4. doi: 10.1001/jama.2016.7653 . 27483067

[49] McCollough C, Cody D, Edyvean S, Geise R, Gould B, Keat N, et al. The measurement, reporting, and management of radiation dose in CT. Report of the American Association of Physicists in Medicine (AAPM) Task Group. 2008;23:1–28. 10.37206/97.

[50] Salimi Y, Shiri I, Akhavanallaf A, Mansouri Z, Saberi Manesh A, Sanaat A, et al. Deep learning-based fully automated Z-axis coverage range definition from scout scans to eliminate overscanning in chest CT imaging. Insights Imaging. 2021; 12(1): 1–16. doi: 10.1186/s13244-021-01105-3 . 34743251 PMC8572075

[51] Malone J, Holmberg O, Simeonov G, editors. Justification of Medical Exposure in Diagnostic Imaging. Proceedings of International Workshop by the International Atomic Energy Agency (IAEA). 2009;79–80. ISSN: 978-92-0-121110-1.

[52] Sakane H, Ishida M, Shi L, Fukumoto W, Sakai C, Miyata Y, et al. Biological Effects of Low-Dose Chest CT on Chromosomal DNA. Radiology. 2020; 295(2): 439–45. doi: 10.1148/radiol.2020190389 . 32154776

[53] Zarb F, Rainford L, McEntee MF. Image quality assessment tools for optimization of CT images. Radiography. 2010; 16(2): 147–53.

[54] Li X, Yang K, Liu B. Exam‐level dose monitoring in CT: Quality metric consideration for multiple series acquisitions. Med Phys. 2019; 46(4): 1575–80. doi: 10.1002/mp.13431 . 30723934

[55] Larson DB, Malarik RJ, Hall SM, Podberesky DJ. System for verifiable CT radiation dose optimization based on image quality. Part II. Process control system. Radiology. 2013; 269(1): 177–85. doi: 10.1148/radiol.13122321 . 23784877

[56] Chun M, Choi YH, Kim JH. Automated measurement of CT noise in patient images with a novel structure coherence feature. Phys Med Biol. 2015; 60(23): 9107. doi: 10.1088/0031-9155/60/23/9107 . 26561914

[57] Chun M, Choi JH, Kim S, Ahn C, Kim JH. Fully automated image quality evaluation on patient CT: Multi-vendor and multi-reconstruction study. PLoS One. 2022; 17(7): e0271724. doi: 10.1371/journal.pone.0271724 . 35857804 PMC9299323

