# Supplementary material for: Development of deep learning-assisted overscan decision algorithm in low-dose chest CT: Application to lung cancer screening in Korean National CT accreditation program
Source: PLoS One. 2022 Sep 29;17(9):e0275531. doi: 10.1371/journal.pone.0275531 (PMC9522252; doi:10.1371/journal.pone.0275531)
Supplement: S1 Table — (DOCX) [file pone.0275531.s001.docx]

**S1 Table.** Detailed scan parameters for external data.

| **No.** | **CT vendor** | **CT machine** | **Reconstruction kernel** | **kVp** | **mAs** | **AEC**^a^ | **Slice thickness  (mm)** |
| --- | --- | --- | --- | --- | --- | --- | --- |
| 1 | Siemens | Spirit | B70s | 130 | 30 | OFF | 1.5 |
| 2 |  | Definition AS+ | I70f | 100 | 50 | OFF | 5 |
| 3 |  | Definition Flash | B70f | 120 | 40$\pm$20 | ON | 1 |
| 4 |  | Definition Flash | I50f | 120 | 15$\pm$6 | ON | 1 |
| 5 |  | Definition AS | B80f | 120 | 12$\pm5$ | ON | 1 |
| 6 |  | Definition AS+ | B60f | 120 | 40 | OFF | 1 |
| 7 |  | Sensation 64 | B60f | 120 | 50 | OFF | 2 |
| 8 |  | Scope | B90s | 110 | 27$\pm$8 | ON | 3 |
| 9 |  | Definition Flash | I41f | 120 | 22$\pm$8 | ON | 2 |
| 10 |  | Definition AS | B80f | 120 | 35 | OFF | 3 |
| 11 |  | Definition AS+ | I50f | 120 | 21 | OFF | 1 |
| 12 |  | Definition AS+ | B60f | 120 | 31$\pm$7 | ON | 1 |
| 13 |  | Sensation 64 | B31f | 120 | 30$\pm$7 | ON | 1.5 |
| 14 |  | Sensation 64 | B70f | 120 | 28$\pm$8 | ON | 1 |
| 15 |  | Definition AS | B80f | 120 | 14$\pm$6 | ON | 1 |
| 16 |  | Definition AS+ | B50f | 100 | 30 | OFF | 3 |
| 17 |  | Sensation 64 | B30f | 120 | 41$\pm$24 | ON | 3 |
| 18 |  | Emotion | B60s | 130 | 36 | OFF | 5 |
| 19 |  | Definition | B60f | 120 | 40 | OFF | 3 |
| 20 |  | Scope | B80s | 110 | 22$\pm$6 | ON | 3 |
| 21 |  | Sensation Open | B60f | 120 | 20 | OFF | 2 |
| 22 |  | Definition AS+ | I70f | 120 | 40 | OFF | 1 |
| 23 |  | Force | Br44f | Sn100^b^ | 167$\pm$106 | ON | 3 |
| 24 | GE | Bright Speed S | STANDARD | 120 | 40$\pm$6 | ON | 1.25 |
| 25 |  | Discovery CT590 RT | LUNG | 120 | 40 | OFF | 1.25 |
| 26 |  | Revolution EVO | LUNG | 120 | 49 | OFF | 5 |
| 27 |  | Discovery CT750 HD | BONE | 120 | 12 | OFF | 1.25 |
| 28 |  | Revolution EVO | LUNG | 120 | 52 | OFF | 1.25 |
| 29 |  | Optima CT660 | BONEPLUS | 120 | 118$\pm$39 | ON | 5 |
| 30 |  | BRIVO CT325 | CHST | 120 | 40 | OFF | 1 |
| 31 |  | Optima CT660 | BONEPLUS | 120 | 26$\pm$12 | ON | 1.25 |
| 32 |  | LightSpeed VCT | LUNG | 120 | 40 | OFF | 2.5 |
| 33 |  | LightSpeed VCT | STANDARD | 120 | 30 | OFF | 1.25 |
| 34 |  | HiSpeed | DETL | 120 | 60 | OFF | 3 |
| 35 | Philips | MX 16 | SA | 120 | 35 | OFF | 1 |
| 36 |  | Brilliance 64 | F | 120 | 45 | OFF | 1 |
| 37 |  | Ingenuity CT | YC | 120 | 30 | OFF | 1 |
| 38 |  | Brilliance 64 | YC | 120 | 30 | OFF | 1.4 |
| 39 |  | Ingenuity Core | C | 120 | 30 | OFF | 1 |
| 40 | Canon | Aquilion ONE | FC86 | 120 | 16$\pm$2 | ON | 2 |
| 41 |  | Aquilion One | FC85 | 120 | 25 | OFF | 1 |
| 42 |  | Aquilion Prime | FC56 | 120 | 25 | OFF | 1 |
| 43 |  | Asteion | FC01 | 120 | 60 | OFF | 2 |
| 44 |  | Asteion | FC53 | 120 | 45 | OFF | 5 |
| 45 |  | Asteion | FC53 | 120 | 50 | OFF | 3 |
| 46 |  | Asteion | FC30 | 120 | 92$\pm$15 | ON | 3 |
| 47 |  | Aquilion Prime | FC55 | 120 | 24 | OFF | 1 |
| 48 |  | Aquilion | FC52 | 100 | 30 | OFF | 1 |
| 49 |  | Aquilion | FC52 | 120 | 69$\pm$14 | ON | 4 |
| 50 |  | Aquilion | FC84 | 120 | 20 | OFF | 3 |

^a^AEC: Automatic exposure control, ^b^Sn100: 100 kVp with tin filter attached.
