# Supplementary material for: Development of deep learning-assisted overscan decision algorithm in low-dose chest CT: Application to lung cancer screening in Korean National CT accreditation program
Source: PLoS One. 2022 Sep 29;17(9):e0275531. doi: 10.1371/journal.pone.0275531 (PMC9522252; doi:10.1371/journal.pone.0275531)
Supplement: S2 Table — The variable consists of age, sex, data source, slices thickness and CT vendors. “Correct” and “Incorrect” indicate the success and fail of overscan detection, respectively. (DOCX) [file pone.0275531.s002.docx]

**S2 Table.** Confusion matrix of each variable in univariate logistic analysis. The variable consists of age, sex, data source, slices thickness and CT vendors. “Correct” and “Incorrect” indicate the success and fail of overscan detection, respectively.

| **Confusion matrix** | | | | | | | |
| --- | --- | --- | --- | --- | --- | --- | --- |
| **Age** | **Correct** | **Incorrect** | **Total** | **Sex** | **Correct** | **Incorrect** | **Total** |
| **Age ≤ 64 yr** | 104 | 2 | 106 | **Male** | 114 | 5 | 119 |
| **Age > 64 yr** | 99 | 5 | 104 | **Female** | 89 | 2 | 91 |
| **Total** | 203 | 7 | 210 | **Total** | 203 | 7 | 210 |
| **Data source** | **Correct** | **Incorrect** | **Total** | **Slice thickness (mm)** | **Correct** | **Incorrect** | **Total** |
| **Internal** | 203 | 7 | 210 | **1 ≤ t < 2** | 230 | 8 | 238 |
| **External** | 48 | 2 | 50 | **2 ≤ t ≤ 5** | 21 | 1 | 22 |
| **Total** | 251 | 9 | 260 | **Total** | 251 | 9 | 250 |
| **Vendors** | **Correct** | **Incorrect** | **Total** |  | | | |
| **Siemens** | 80 | 3 | 83 |  |  |  |  |
| **GE** | 68 | 3 | 71 |  |  |  |  |
| **Philips** | 63 | 2 | 65 |  |  |  |  |
| **Canon** | 40 | 1 | 41 |  |  |  |  |
| **Total** | 251 | 9 | 260 |  |  |  |  |
