# Supplementary material for: Development of deep learning-assisted overscan decision algorithm in low-dose chest CT: Application to lung cancer screening in Korean National CT accreditation program
Source: PLoS One. 2022 Sep 29;17(9):e0275531. doi: 10.1371/journal.pone.0275531 (PMC9522252; doi:10.1371/journal.pone.0275531)
Supplement: S3 Table — (DOCX) [file pone.0275531.s003.docx]

**S3 Table.** Excessive effective doses caused by overscans for internal and external dataset.

| Internal data | | | | | | |
| --- | --- | --- | --- | --- | --- | --- |
| No | kVp | mAs | Slice thickness (mm) | CTDI_vol_^b^  (mGy) | Overscan length  (cm) | Excessive effective dose  (mSv) |
| 1 | Sn100^a^ | 150 | 1 | 0.519 | 2.3 | 0.00704 |
| 2 | 120 | 30 | 1 | 2.028 | 2.9 | 0.03469 |
| 3 | 120 | 30 | 1 | 2.028 | 2.1 | 0.02512 |
| 4 | 120 | 30 | 1 | 2.028 | 2.4 | 0.02871 |
| 5 | 120 | 30 | 1 | 2.028 | 2.1 | 0.02512 |
| 6 | 120 | 30 | 1 | 2.028 | 2.7 | 0.03230 |
| 7 | 120 | 30 | 1 | 2.028 | 3.6 | 0.04306 |
| 8 | 120 | 30 | 1 | 2.028 | 1.5 | 0.01794 |
| 9 | 120 | 30 | 1 | 2.028 | 0.9 | 0.01077 |
| 10 | 120 | 30 | 1 | 2.028 | 1.4 | 0.01675 |
| 11 | 120 | 30 | 1 | 2.028 | 2.5 | 0.02991 |
| 12 | 120 | 30 | 1 | 2.028 | 1 | 0.01196 |
| 13 | 120 | 30 | 1 | 2.028 | 1.5 | 0.01794 |
| 14 | 120 | 30 | 1 | 2.028 | 0.9 | 0.01077 |
| 15 | 120 | 30 | 1 | 2.028 | 1 | 0.01196 |
| 16 | 120 | 38 | 1.25 | 3.060 | 1.25 | 0.02257 |
| 17 | 120 | 38 | 1.25 | 3.060 | 1.25 | 0.02257 |
| 18 | 120 | 38 | 1.25 | 3.060 | 0.75 | 0.01354 |
| 19 | 120 | 38 | 1.25 | 3.060 | 1.5 | 0.02708 |
| 20 | 120 | 58 | 1.25 | 3.060 | 1.875 | 0.03385 |
| 21 | 120 | 58 | 1.25 | 3.060 | 3.75 | 0.06770 |
| 22 | 120 | 38 | 1.25 | 3.060 | 1.25 | 0.02257 |
| 23 | 120 | 38 | 1.25 | 3.060 | 1.375 | 0.02482 |
| 24 | 120 | 25$\pm$2 | 1.25 | 1.140 | 1.875 | 0.01261 |
| 25 | 120 | 25$\pm$2 | 1.25 | 1.120 | 1.25 | 0.00826 |
| 26 | 120 | 19$\pm$4 | 1.25 | 0.890 | 1.875 | 0.00985 |
| 27 | 120 | 27$\pm$1 | 1.25 | 1.180 | 1.25 | 0.00870 |
| 28 | 120 | 25$\pm$2 | 1.25 | 1.130 | 1.25 | 0.00833 |
| 29 | 120 | 22$\pm$3 | 1.25 | 1.020 | 1.375 | 0.00827 |
| 30 | 120 | 20$\pm$4 | 1.25 | 0.900 | 1.125 | 0.00597 |
| 31 | 120 | 20 | 1 | 1.700 | 1.5 | 0.01505 |
| 32 | 120 | 20 | 1 | 1.700 | 1 | 0.01003 |
| 33 | 120 | 20 | 1 | 1.700 | 1 | 0.01003 |
| 34 | 120 | 30 | 1 | 2.000 | 1.5 | 0.01770 |
| 35 | 120 | 30 | 1 | 2.000 | 1.8 | 0.02124 |
| 36 | 120 | 30 | 1 | 2.000 | 2.2 | 0.02596 |
| 37 | 120 | 30 | 1 | 2.000 | 1.9 | 0.02242 |
| 38 | 120 | 30 | 1 | 2.000 | 2.2 | 0.02596 |
| 39 | 120 | 30 | 1 | 2.000 | 1 | 0.01180 |
| 40 | 120 | 30 | 1 | 2.000 | 1.7 | 0.02006 |
| 41 | 120 | 30 | 1 | 2.000 | 0.6 | 0.00708 |
| 42 | 120 | 30 | 1 | 3.300 | 1 | 0.01947 |
| 43 | 120 | 30 | 1 | 3.100 | 1 | 0.01829 |
| 44 | 120 | 108$\pm$22 | 1 | 9.400 | 1 | 0.05546 |
| 45 | 120 | 30 | 1 | 3.100 | 1.2 | 0.02195 |
| 46 | 120 | 96$\pm$26 | 1 | 9.900 | 1 | 0.05841 |
| 47 | 120 | 30 | 1 | 3.100 | 1.2 | 0.02195 |
| External data | | | | | | |
| No | kVp | mAs | Slice thickness (mm) | CTDI_vol_^b^  (mGy) | Overscan length  (cm) | Excessive effective dose  (mSv) |
| 1 | 100 | 50 | 5 | 1.971 | 1 | 0.01163 |
| 2 | 120 | 40$\pm$20 | 1 | 3.454 | 0.9 | 0.01834 |
| 3 | 120 | 15$\pm$6 | 1 | 2.304 | 0.6 | 0.00816 |
| 4 | 120 | 40 | 1 | 2.720 | 1.4 | 0.02247 |
| 5 | 120 | 40 | 1.25 | 3.120 | 1.25 | 0.02301 |
| 6 | 110 | 27$\pm$8 | 3 | 2.371 | 6.3 | 0.22406 |
| 7 | 120 | 35 | 3 | 2.660 | 2.7 | 0.04238 |
| 8 | 120 | 52 | 1.25 | 1.150 | 1.25 | 0.00848 |
| 9 | 120 | 118$\pm$39 | 5 | 2.960 | 2.5 | 0.04366 |
| 10 | 120 | 26$\pm$12 | 1.25 | 3.110 | 0.625 | 0.02916 |
| 11 | 120 | 45 | 1 | 2.900 | 0.6 | 0.01027 |
| 12 | 100 | 30 | 1 | 3.000 | 1.4 | 0.02478 |
| 13 | 120 | 30 | 1.4 | 2.000 | 1.68 | 0.01982 |
| 14 | 120 | 20 | 3 | 2.900 | 0.6 | 0.01027 |
| 15 | 120 | 40 | 1 | 2.699 | 1.3 | 0.02070 |
| 16 | 100 | 167$\pm$106 | 3 | 1.337 | 2.4 | 0.01893 |

^a^Sn100: 100 kVp with tin filter attached. ^b^CTDI_vol_ : Volume CT dose index
