# Supplementary figures and images for: Development of deep learning-assisted overscan decision algorithm in low-dose chest CT: Application to lung cancer screening in Korean National CT accreditation program
Source: PLoS One. 2022 Sep 29;17(9):e0275531. doi: 10.1371/journal.pone.0275531 (PMC9522252; doi:10.1371/journal.pone.0275531)

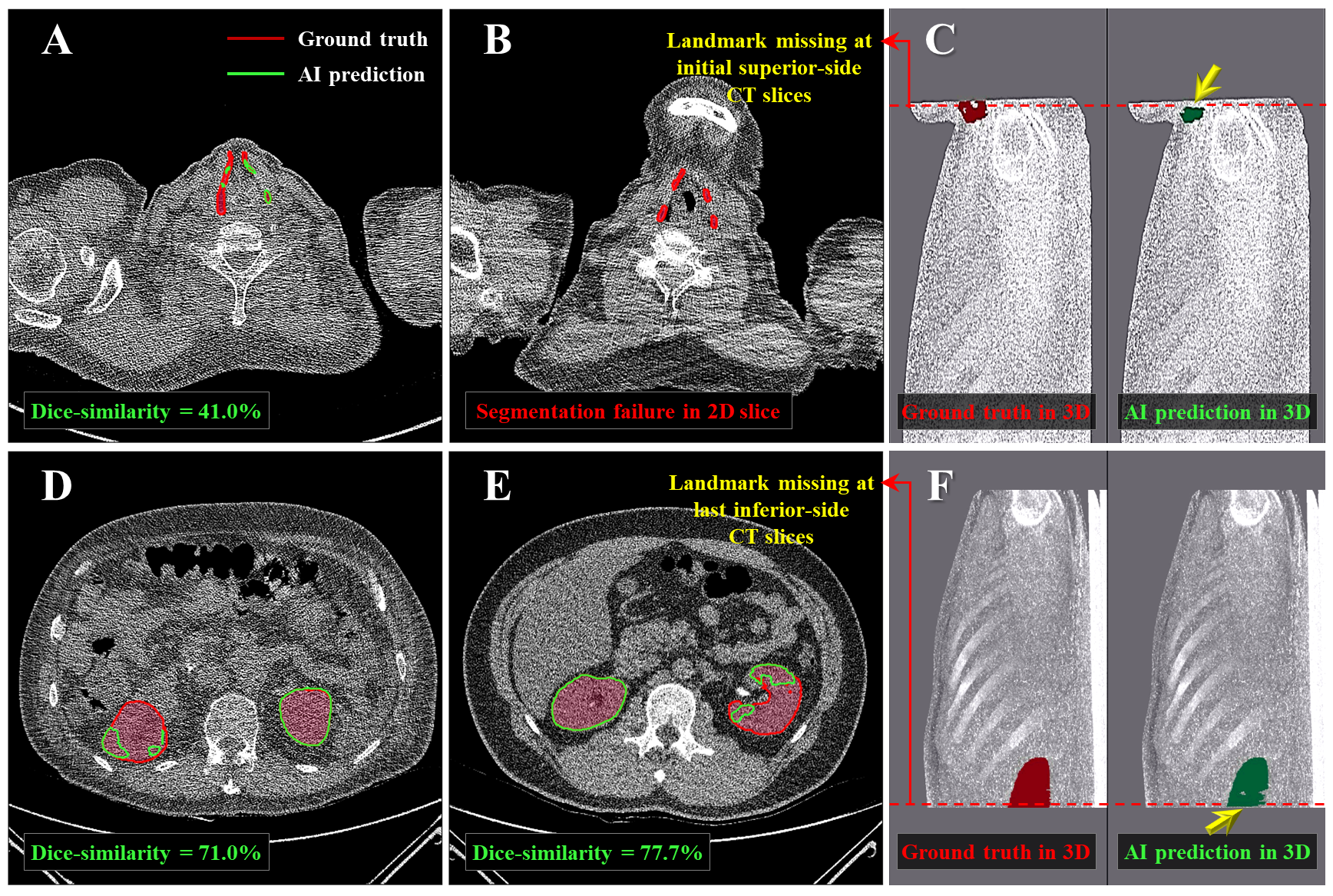

Supplement: S1 Fig — Segmentation results for superior-side (A-C) and inferior-side (D-F) were visualized in 2D axial view and 3D rendering view. The use of only two-dimensional information on landmarks’ existence may exhibit an entire (B) or partial (E) segmentation failure, while the use of volumetric information stacked from 2D information has been improved in landmark detections (C, F). Ground truth label and AI predictions were marked as red and green lines, respectively. (W/L = 400/40 HU). (TIF) [file pone.0275531.s004.tif]
