# Supplementary material for: Development of deep learning-assisted overscan decision algorithm in low-dose chest CT: Application to lung cancer screening in Korean National CT accreditation program
Source: PLoS One. 2022 Sep 29;17(9):e0275531. doi: 10.1371/journal.pone.0275531 (PMC9522252; doi:10.1371/journal.pone.0275531)
Supplement: Attachment — Submitted filename: Response_to_Reviewers_v1.6.docx [file pone.0275531.s005.docx]

**Response to Review Comments**

Journal: PLoS One

Article reference: PONE-D-22-05608

Author(s): Sihwan Kim; Woo Kyoung Jeong; Jin Hwa Choi; Jong Hyo Kim; Minsoo Chun

Title: Development of Deep Learning-Assisted Overscan Decision Algorithm in Low-dose Chest CT: Application to Lung Cancer Screening in Korean National CT Accreditation Program.

Dear editors:

We are thankful to the editor and reviewers for their valuable comments on our previous manuscript. We have carefully considered reviewers’ comments in preparing our revision. Our responses to the comments and suggestions are provided below (the reviewers’ comments are with standard font and our responses are with blue color). Modifications according to the reviewers’ and the editor’s comments are marked with blue color in the revised manuscript. We slightly changed the title of submitted paper.

**Response to comments of the reviewer #1**

1. This paper proposed an AI-based target organ segmentation (plus logical operation) method to automatically determine the overscan of LDCT of chest screening. The authors used a relatively small data set (without details on the patient cohort information, age, size, only mentioned sex in the results).

🡺 Thanks for the comment. As you pointed out, the number of datasets is relatively small because the all data were collected from external audit executed by KIAMI. To overcome the relatively small number of data, we augmented the training data with random rotating and translation in each training epoch. Also, we specified the demographics and scan parameters of the CT images used for validation of AI-based segmentation and overscan algorithm, which are summarized in Table 1.

2. Main concern of reviewer #1 is on the handling of the underscan. As the authors stated in the introduction, underscan actually has more negative dose concerns than the overscan if a new scan has to be repeated. But in the current study, underscan is also considered as “pass”. I understand that for underscan you might not be able to detect the target organ slices, or only part of them, using the AI. But equal effort should be spent on the underscan if the purpose is to avoid extra dose.

🡺 Thanks for the comment. I agree with your opinion that it is important to prevent the repetitive scans caused by the an underscan. Thus, we partially amended the stage 2 and 3 of the overall algorithm (without compromising its performance) to utilize the underscan information (Fig. 2). The revised algorithm could simultaneously give alerts on both the overscan and the underscan. Revised information is reflected on the section of ‘materials and method’ as follow;

[line 137-141]

“In the second stage, there are two determination branches according to the landmark types. Each branch comprised of rule-based logical operations, reflecting the human decision-making process. The identification information (information A-D in Fig 2) of overscan/underscan was logically acquired for each determination branch, based on the information of predefined landmark presence and its relative position to initial/last CT slice.”

[line 147-151]

“While the overscan decision was made when the length exceeded the threshold (τ*_superior_*), the algorithm further examined whether the patches contained the lung parenchymal tissue if the length was less than the threshold. The decision was made as appropriate scan range or underscan according to the existence of the lung parenchymal tissue within the patches.”

[line 161-165]

“If the last inferior-side ROI patch included the region of the lung parenchyma, it was concluded as the inferior underscan. Scan range identification information (Fig 2, information A-D), as the results for logical operation stage, were set as ‘False’ at default. When the overscan or underscan occurred, the Boolean value was converted from ‘False’ to ‘True’. The final Boolean values in information A-D were transmitted to the last decision stage.”

However, the main scope of our study is limited to events of the overscan, and as the number of cases of underscan currently available is extremely limited (0.4%), it is judged that a separate study will be necessary for the evaluation on the underscan, as the next research topic. We wrote this sentence as the limitation in ‘Discussion’ section a below;

“Finally, there exists only few underscan cases (0.4%), and the separate study to evaluate the underscan is necessary as a future research topic.”

3. Following the above comment, would suggest the authors report on the dose increases in real values for overscan/underscans they detected. This will put the value of this study into content. How much of dose increases are we detecting?

🡺 Thanks for the comment. It is quite better to suggest the report of dose increase for overscan/underscan cases, but the number of the underscan is only the one. Thus, we mainly focused on the increased dose of the overscan, and added the Table 4 and corresponding description in ‘Materials & Methods’, ‘Results’ and ‘Discussion’.

[Materials & Methods]

“The excessive effective dose was estimated by multiplying volume CT dose index (CTDI_vol_), overscan length and the tissue conversion factor (*k*). The CTDI_vol_ was obtained from either CT DICOM header and patient’s dose report. The *k* value was referenced from the report of American Association of Physicists in Medicine (AAPM) [49] and was 0.0059 and 0.015 for the superior and inferior-side overscan, respectively, which corresponds to neck and abdomen region. The detailed scan parameters and the excessive effective dose was provided in S3 Table.”

[Results]

“Detailed scan parameters and estimated excessive effective doses for overscan data were provided in S3 Table. The mean CTDI_vol_ of overscan cases was 2.42 ± 1.69 mGy and 2.54 ± 0.65 mGy, and the mean overscan length was 1.59 ± 0.7 cm and was 1.66 ± 1.41 cm for internal and external data, respectively. The estimated effective doses caused by the overscan increased about 0.02 ± 0.01 mSv and 0.03 ± 0.05 mSv for internal and external data, respectively.”

[Discussion]

“Considering the mean effective dose being about 0.02 mSv in case of plain PA chest radiograph, the estimated excessive dose in our study are equivalent or 1.5 times greater than those in chest PA on average [51]. In the worst overscan case, the excessive effective dose caused by overscan could reach up to 11 times of those by plain PA chest radiograph, which cannot be a negligible level. We hope that our automatic overscan decision algorithm contribute to precisely identify excessive overscan dose and provide more quantitative guidelines on minimizing unnecessary scan ranges in an institutional monitoring.”

4. Another follow-up question is that it is not clear to me exactly how the target organ information is used after detection, if there’s space, more figures/graphs showing the exact boundary of the organs in regard to a specific scan-range will be very helpful.

🡺 Thanks for the comments. Not to give a confusion to readers, we added following figure as Fig 3 and described it with exact organ boundaries.

**Fig 3**. **Sample landmark segmentation comparison between AI-assisted model and the experienced radiologist.** The results were visualized at different scanning position; (A) segmentation result of superior-side scan at overscan position, (B) that of superior-side scan at acceptable scan position, and (C, D) that of inferior-side scan at acceptable scan position. Ground truth label and AI-model’s prediction result were displayed with red and green lines, respectively. (W/L: 400/40).

5. Line 67-69, redundant statement as Line 55-57. Only need to keep one place.

🡺 Thanks for the comments. Not to give a confusion to readers, we removed the redundant sentence of Line 55-57 in the section of ‘Introduction’ and amended the paragraph as follows;

“Although computed tomography (CT) technology has developed and achieved outstanding diagnostic accuracy, there remains concerns regarding radiation-induced cancers driving an as low as reasonably achievable (ALARA) movement [1-3]. The adoption of a low-dose CT (LDCT) protocol and the scan range optimization are exemplary efforts to lower imaging doses [4-10]. Particularly, LDCT has been popularly used in lung cancer screening program enabling to detect cancers in early stage, thereby reducing mortality rates [11-12]. While the LDCT protocol has been adopted with various aspects, to the best of our knowledge, scan range selections still rely on a manual decision by the radiation technologist exhibiting intra- and inter-institution variations [13-19]. However, as a manual range selection is vulnerable to prevent excessive patients’ doses, efforts should be made to provide optimal scan range and reduce inter-individual variability. Whereas excessive scan may increase unnecessary doses, the worse scenario is to scan with insufficient coverages requiring additional examination [20-22]. Both situations necessitate the scan range monitoring procedure either prospective or retrospective way.”

6. Line 257-258, it seems from Table 3 that the p values are the numbers determined, not a range, should be something like, p = 0.431, not p<0.431. Please double check.

🡺 Thanks for the comments. we amended the sentence as follows;

“Table 3 shows the results of univariate logistic regression analysis. The univariate analysis revealed that age, sex, data source, CT vendor, and slice thickness had no statistically significant influence on the algorithm’s decision.”

7. For all the statistical results with CI, not sure why quite some values are very skewed, i.e. not close to the center of the CI. For example, Table 3, Odds ratio, typically should be symmetric for the estimated value within the CI. And Table 2, sensitivity for external data, with such a large CI, 69% to 99%, the estimated value is still 93%? Can you double check?

🡺 Thanks for the comments. The main reason for asymmetric distribution of odds ratio’s C.I. is related with a log transformation process. When computing the odd ratio, we use the log transformation because the odds ratio is not normally distributed. Hence, the C.I. becomes an asymmetric by using natural log (LN) of the odds ratio and its anti-log process for the calculation of lower-limit and upper-limit of C.I. for odds ratio [Knowledge Ref. 1].

[Knowledge Reference 1] Lisa Sullivan, PhD et al, Class Material for Biostatistics “Confidence Intervals: Computing the confidence interval for an odds ratio”, weblink: https://sphweb.bumc.bu.edu/otlt/mph-modules/bs/bs704_confidence_intervals/bs704_confidence_intervals_print.html

🡺 The reason for wide and asymmetric C.I. of sensitivity is related on the calculation method of the C.I.; "exact" Clopper-Pearson confidence intervals. The sensitivity, specificity and accuracy are the proportion, thus the according confidence intervals can be calculated by using standard methods for proportions [Knowledge Ref. 2]. When constructing the C.I. of proportion from a 2 x 2 confusion matrix, the binomial distribution is generally used. Thus, the Clopper-Pearson interval was used for exact estimating of the C.I., not the normal approximation method (which assumes the normal distribution and has the symmetric interval). When using it, the only case of a symmetric C.I. is when the probability of the proportion becomes 0.5 [Knowledge Ref. 3, 4]. The larger C.I. compared to the normal approximation interval is the characteristic of Clopper-Pearson interval. If the sampling number (n) increases enough, the C.I. would be narrower and looks more symmetric.

[Knowledge Reference 2] Wen Zhu et al, “Sensitivity, Specificity, Accuracy, Associated Confidence interval and ROC Analysis with Practical SAS Implementations” weblink: https://lexjansen.com/nesug/nesug10/hl/hl07.pdf

[Knowledge Reference 3] “Binomial Proportion Confidence Interval on Wikipedia”, weblink: https://en.wikipedia.org/wiki/Binomial_proportion_confidence_interval#Clopper%E2%80%93Pearson_interval

[Knowledge Reference 4] “Binomial Confidence Interval Estimation – Why is not symmetric?”, weblink: https://stats.stackexchange.com/questions/4713/binomial-confidence-interval-estimation-why-is-it-not-symmetric

8. S1 Table, for Philips scanner, iDose is the iterative recon strength level, not the recon kernel. Same problem for S2 Table, ASiR-V is the algorithm, not the kernel. On the opposite side, S3 Table 3, all the recon kernels were correctly reported, but under the “reconstruction method” column. Please correct those.

🡺 Thanks for the comments. Not to give confusion to readers, we unified the all confusing terms as the ‘reconstruction kernel’ for all suggested Tables.

**Response to comments of the reviewer #2**

1. As the ultimate goal is to reduce radiation exposure for the benefit of the patients, I'd like to know the reasons why the authors chose to focus on monitoring the scan range retrospectively instead of predicting the appropriate scan range before the scan.

🡺 Thanks for the comments. Recent studies have shown attempts to automatically define the scan-range on topogram based on AI technology to give objectivity to scan-range setting before the primary CT scan. However, concerns about unverified AI technologies for untrained data from different CT machines (or hospitals) still make it difficult to rule out CT operator’s final intervention. In this study, to solve the overscan problem by reflecting the realistic clinical workflow, we focused on minimizing the operator’s error themselves through a retrospective auditing rather than approaching it from the perspective of automatic range definition. In other words, the significance of this study is to reduce an uncertainty of the scan range retrospectively and gradually through the AI-assisted overscan audit procedure.

2. Theoretically, the method of deep learning segmentation and using landmarks to identify scan range can be applied to topograms as well. If topograms were used, they could potentially be used to prevent overscan. What is the main reason to use axial slices over topograms besides the lack of images?

🡺 Thanks for the comments. We agreed with the importance of dose optimization through the pre-scan range recommendation based on topogram, but it still required further technical validation on applying it to clinical workflows. However, the purpose of our study is not a scan range recommendation, but a retrospective scan range audit. The main reason for conducting an audit in axial slices is to use clear landmark boundary contours, unavailable on the topogram limited by superimposed pixel intensity. Thus, we reflect related concerns in ‘Discussion’ section as follows;

“The purpose of our study was to develop a deep learning-assisted algorithm to discriminate the overscan cases of lung cancer screening program in LDCT scan. Most of the previous studies on overscan determination [10, 18] in chest CT scans mainly focused on scan range delimitation in topograms, but not in axial CT slices. Automatic scan range delimitation in the topogram domain would be a meaningful approach because the topogram defined the regions being scanned prior to actual scans. In this regards, scan range recommendations in topogram stage enable to reduce the workload and inter-operator variability. and there have been various related researches. However, it is challenging and premature because there are opaque shadings and intensity variation across CT vendors in topograms, and even AI-assisted approaches are still hard to localize key landmarks [22]. Furthermore, localization comparison between scout and axial images should be preceded for their clinical routine [50]. Although optimal scan range recommendations prior to CT scans is meaningful, we mainly focused to establish more objective system to retrospectively assess the overscan length and its corresponding excessive doses and there are no previous related studies.”

3. When presenting the segmentation results, please provide example images segmented by the DL algorithm and the radiologist.

🡺 Thanks for the comments. We added visualized results comparing the segmentation of DL algorithm and that of radiologist in the Fig 3.

4. Please provide the confusion table before you calculate the accuracy, sensitivity, specificity, PPV, and NPV in Table 2, since all those metrics came from the same confusion table. Please provide the contingency table for your Fisher's exact test as well. Similarly, provide the contingency tables for your univariate OR analysis as an addition to Table 3.

🡺 Thanks for the comments. we added each contingency table of decision model validation in Table 2 and that of univariate OR analysis in S2 Table (in supporting information). The Fisher’s exact test shares the same contingency table with decision model validation (Table 2).

5. It is good to see the univariate analysis in Table 3. But it is also important to know when your automated tool fails, especially when a deep learning algorithm is involved. Therefore, I'd love to see an error analysis. You have acknowledged that segmentation may affect it. If so, please add a couple of image examples along with the dice scores to Discussion. If the segmentation performance is correlated with any of the variables listed in Table 3, please add the dice scores to Table 3 as well.

🡺 Thanks for the comments. To give a better understanding to readers, we add error analysis of AI-assisted segmentation model to ‘Discussion’ with the supporting Information as ‘S4 Fig’ as follows;

“First, in the AI model segmentation stage, there might exist segmentation errors for both superior and inferior direction (S4 Fig). The main reasons were extreme quantum-noise of LDCT and lack of a diversity of trained data distribution. However, despite the segmentation errors, the developed algorithm minimized its vulnerability to error cases by aggregating the 2D segmented landmark information into 3D volumetric information. Furthermore, several segmentation errors could be filtered out and improved by the logical operation of the second-stage.”

**S4 Fig.** Error examples for AI-assisted segmentation model. Segmentation results for superior-side (A-C) and inferior-side (D-F) were visualized in 2D axial view and 3D rendering view. The use of only two-dimensional information on landmarks’ existence may exhibit an entire (B) or partial (E) segmentation failure, while the use of volumetric information stacked from 2D information has been improved in landmark detections (C, F). Ground truth label and AI predictions were marked as red and green lines, respectively. (W/L = 400/40 HU).

However, we had to consider that calculation of the Dice-coefficient with respect to variables of Table 3 was limited to be conducted as the labeled mask of the landmark organs (e.g. thyroid cartilage or kidney) by an experienced-radiologist was not available in performance evaluation dataset.

6. The figure and table captions are not self-explanatory. Please explain what's in the figure/table in the caption with more details.

🡺 Thanks for the comments. For a better understanding, we amended the captions of figure/table as follows;

**Fig 1.** **Representative landmarks in overscan decision.** (A) Vocal cord as an initial superior-side landmark, (B) Thyroid cartilage as a replaced superior-side landmark, (C) Kidney as an inferior-side overscan landmark.

**Fig 2.** **Schematic diagram of overscan determination process.** Stage 1: AI-assisted landmark segmentation. Stage 2: Rule-based logical operations. Stage 3: Final overscan decision and its alerts.

**Fig 3. Sample landmark segmentation comparison between AI-assisted model and the experienced radiologist.** The results were visualized at different scanning position; (A) segmentation result of superior-side scan at overscan position, (B) that of superior-side scan at acceptable scan position, and (C, D) that of inferior-side scan at acceptable scan position. Ground truth label and AI-model’s prediction result were displayed with red and green lines, respectively. (W/L: 400/40).

**Fig 4.** ROC curve and AUROC values for internal (A) and external data (B), respectively.

**Table 1.** **Details on internal data.** Demographics and scan parameters are presented according to the data usage.

**Table 2.** Contingency table of decision model validation with internal and external data.

**Table 3****.** **Univariate logistic regression analysis**. The internal and external data were rearranged to satisfy pre-defined univariate condition for each variable.

7. Typo on line 242: The accuracy of internal data ranges from 93.24% while the number is 93.25% in Table 2.

🡺 Thanks for the comments. We amended the typo from ‘93.24%’ to ‘93.25%’ in the ‘Results’ section as follows;

“In decision model validation, the accuracy of the algorithm was 96.67 (95% CI: 93.25%-98.65%) and 96.00 (95% CI: 86.29%-99.51%) for internal and external data, respectively.”

**Response to comments on journal requirements and the associate editor**

1. Please ensure that your manuscript meets PLOS ONE's style requirements, including those for file naming.

🡺 Thanks for your recommendation. We modified the manuscript by following PLOS One template and all figures were validated with PACE system.

2. We note that the grant information you provided in the ‘Funding Information’ and ‘Financial Disclosure’ sections do not match. When you resubmit, please ensure that you provide the correct grant numbers for the awards you received for your study in the ‘Funding Information’ section.

🡺 Thanks for your guidance. We have checked the grant numbers.

3. Please remove any funding-related text from the manuscript and let us know how you would like to update your Funding Statement. Currently, your Funding Statement reads as follows:

“This work was supported by the Korea Medical Device Development Fund grant funded by the Korean government (the Ministry of Science and ICT, the Ministry of Trade, Industry and Energy, the Ministry of Health & Welfare, the Ministry of Food and Drug Safety) (Project Number: 1711138600, 1711138601, KMDF_PR_20200901_0267).”

Please include your amended statements within your cover letter; we will change the online submission form on your behalf.

🡺 Thanks for the comments. We removed any funding-related statement in other areas of the manuscript except in funding statement area. We will keep same funding statements in the cover letter from initial submission.

4. We note that you have stated that you will provide repository information for your data at acceptance. Should your manuscript be accepted for publication, we will hold it until you provide the relevant accession numbers or DOIs necessary to access your data. If you wish to make changes to your Data Availability statement, please describe these changes in your cover letter and we will update your Data Availability statement to reflect the information you provide."

🡺 Thanks for the comments. We will keep a same opinion with those at initial submission. I will provide repository information for data at acceptance.

We again sincerely appreciate for the valuable comments and reviewing our manuscript.

Yours sincerely,

Minsoo Chun, Ph.D.
