# Supplementary material for: Development of deep learning-assisted overscan decision algorithm in low-dose chest CT: Application to lung cancer screening in Korean National CT accreditation program
Source: PLoS One. 2022 Sep 29;17(9):e0275531. doi: 10.1371/journal.pone.0275531 (PMC9522252; doi:10.1371/journal.pone.0275531)
Supplement: Attachment — Submitted filename: Response_to_Reviewers_v2.1.docx [file pone.0275531.s006.docx]

**Response to Review Comments**

Journal: PLoS One

Article reference: PONE-D-22-05608

Author(s): Sihwan Kim; Woo Kyoung Jeong; Jin Hwa Choi; Jong Hyo Kim; Minsoo Chun

Title: Development of Deep Learning-Assisted Overscan Decision Algorithm in Low-dose Chest CT: Application to Lung Cancer Screening in Korean National CT Accreditation Program.

Dear editors:

We are thankful to the editor and reviewers for their valuable comments on our previous manuscript. We have carefully considered reviewers’ comments in preparing our revision. Our responses to the comments and suggestions are provided below (the reviewers’ comments are with standard font and our responses are with blue color). Modifications according to the reviewers’ and the editor’s comments are marked with blue color in the revised manuscript.

**Response to comments of the reviewer #1**

1. Fig. 3, very nice visualization. Just why there are two acceptable locations for the inferior side (C and D)? Need a little more explanation in the text. I saw some descriptions on the supplemental materials about the AI failing in 2D. But still not clear why there are two acceptable positions shown in Fig. 3. Also, do you have any over scan images for the inferior side?

🡺 Thanks for the comment. The two acceptable locations, which may be confusing, were reduced to one (Fig. 3C), and an example image of inferior-side overscan was added (Fig. 3D). Followed by the revised figure, corresponding figure caption was amended.

**Fig 3.** **Sample landmark segmentation comparison between AI-assisted model and the experienced radiologist.** The results were visualized at different scanning position; (A) segmentation result of superior-side scan at overscan position, (B) that of superior-side scan at acceptable scan position, (C) that of inferior-side scan at acceptable scan position, and (D) inferior-side scanning example at overscan position. Ground truth label and AI-model’s prediction result were displayed with red and green lines, respectively. (W/L: 400/40).

2. The overall additional dose from overscan. As I have expected, the mean added effective dose due to overscan is really low. 0.02 mSv should not be any concern when the opposite side is underscan with missed information. I see that the authors tried to defend that the dose is not low in order to justify the value of the study. I kind of disagree with that logic. It is good news that after this study, we found out that the dose from overscan is really low, not to any concern. The study is still valuable. It is not necessary to overstate the dose issue to justify the study.

🡺 Thanks for the comment. We agree with the emphasis on additional effective dose by the overscan at low dose CT. Thus, we amended the analysis on excessive effective dose as below;

“Considering the mean effective dose being about 0.02 mSv in case of plain PA chest radiograph, the estimated excessive effective dose in our study are equivalent or 1.5 times greater than those in chest PA on average [51]. In the worst overscan case of the study, the excessive effective dose caused by overscan could reach up to 11 times of those by plain PA chest radiograph. However, all those effective dose levels were much below the possible chromosomal damage level (5 mSv) by the X-ray radiation [52]. In the case of slight deviations from the acceptable scan range, especially for LDCT scan, it is thought that excessive effective dose is not a concern.”

**Response to comments of the reviewer #2**

1. In this revision, the authors clarified that the purpose of the work is to reduce subjectivity of auditing of overscans (by radiologists) and save time/cost/resources. However, the gold standard used in this study was from only 1 radiologist.

🡺 Thanks for the comment. As you pointed out, creating a gold standard by reflecting the opinions of more than two experts would help secure experiment’s objectivity. Although the study’s gold standard was recorded by the experienced radiologist, it could be affected by a subjective bias. However, further gold standard recording is practically limited in this study. Hence, we added it as the limitation in ‘Discussion’ section as follows.

“First, a subjective bias could affect the gold standard, even though it was created by an experienced radiologist. In the future study, it might better to secure an objectivity by utilizing records of more than two experts.”

2. If the authors wish to reduce the radiologists' burden to audit overscans, it is probably better to completely eliminate the false negatives (algorithm = None, radiologist = Overscan) so that the radiologists do not need to review every single non-overscan cases to confirm that 1 false negative (according to Table 2, there is 1 with internal data and 1 with external data). Therefore, I suggest the authors do a more in-depth analysis on why the false negatives occurred and maybe also tune your network to prioritize the elimination of false negatives even if it means the false positive cases (algorithm = Overscan, radiologist = None) may increase greatly. Note that this comment is based on my assumption that overscans are rare compared to appropriate scan range and that no overscan case can be missed (therefore the review by radiologist).

🡺 Thanks for the comment. The reason for false-negative (algorithm = None, radiologist = Overscan) occurrences for both datasets was that the length of the transition area in superior-side scan was not over the pre-defined overscan cut-off threshold (τ). Although our algorithm could force false-positive to increase and make the false-negative zero by adjusting the cut-off threshold, I don’t think it’s the best idea due to the occurrence frequency of the overscan. In retrospective audit process, the more false-positive alerts, the more workload radiologist have. As we described the overscan rate in Dataset paragraph of ‘Materials and Methods’ section, it occurred at a frequency of 22.4% and 32% for internal and external dataset, respectively. In my opinion, occurrence frequency more than 20% is not the rare. It means that false-positive is also a condition that must be considered.

We added the related statement in the limitation paragraph of the ‘Discussion’ section as below;

“Third, analyzing the contingency table in both internal and external data, the algorithm still had a few false-negatives (algorithm = none, radiologist = overscan) and false-positives (algorithm = overscan, radiologist = none). The reason for false-negative occurrences in both data was that the length of the transition area in superior-side scan was not over the pre-defined overscan cut-off threshold. In most of the false-positive cases, the partial segmentation failure of the landmark at the initial or last slice position was considered as a main reason in our algorithm. Although our algorithm could coordinate false-positives and false-negatives by adjusting the cut-off threshold (τ*_superior_* or τ*_inferior_*), the optimal threshold might require a large scale and multi-centre investigation.”

3. I assume that the every overscan case decided by the algorithm would be reviewed by radiologists so that no operator would be wrongfully accused. I am not sure how often/rare overscan occurs in routine LDCT lung scans, but the authors can probably provide an estimate on how much time it could save by only having to review the overscans brought up by the algorithm. I believe the algorithm proposed by the authors is potentially useful. However, I do not think the authors have explained how to realistically help reduce the burden of auditing overscans on radiologists. Just having an algorithm that has good overall accuracy is not enough because radiologists would still need to review all the cases.

🡺 Thanks for the precise comments. As you pointed out, by using the developed algorithm, the radiologists only needed to pay attention to the alerting cases by the overscan/underscan. In overscan cases, it occurred in internal and external datasets at a frequency of 22.4% and 32%, respectively. Accordingly, if CT scan data was examined for all patients, it was estimated that the developed algorithm could reduce the workload of overscan range check by 68% and 77.6% for each studied dataset.

Including above statements, we added the detail descriptions on the practical effects from reducing the burden of auditing overscans in ‘Discussion’ section, and also described further applications creating a clinical impact.

“Beyond limitations, by using the developed algorithm, the radiologists only need to pay attention to the alerting cases by the overscan/underscan. The less overscan rate, the less radiologist’s workload could be accomplished. In comparison with every single patient audit procedure, as the overscan occurred in internal and external datasets at a frequency of 22.4% and 32%, it was estimated that the developed algorithm could reduce the workload of overscan range check by 68% and 77.6% for each dataset. We also expect that combinations of our algorithm with radiation dose and image quality monitoring could establish the fully automated and integrated CT quality system for every patient [52-56]. By reducing human and time resources with full automation, it is available to equip high throughput and objective quality monitoring platform for the entire CT scans.”

We again sincerely appreciate to reviewers and editors for the valuable comments and reviewing our manuscript.

Sincerely yours,

Minsoo Chun, Ph.D.
